# Draft assemblies for three geographically distinct isolates of the human-infecting microsporidium *Encephalitozoon intestinalis*

**DOI:** 10.1128/mra.00933-25

**Published:** 2025-11-04

**Authors:** Alexander Thomas Julian, Sunah Lee, Paulina Markiv, Anne Caroline Mascarenhas dos Santos, Jialing Xiang, Jean-Francois Pombert

**Affiliations:** 1 Department of Biology, Illinois Institute of Technology 2455, Chicago, Illinois, USA; 2 Biosciences and Biotechnology Division, Lawrence Livermore National Laboratory 4578, Livermore, California, USA; University of California Riverside, Riverside, Riverside, USA

**Keywords:** *Encephalitozoon*, computational biology, genomes, intracellular parasites, microsporidia

## Abstract

*Encephalitozoon intestinalis* is an obligate intracellular parasite that causes enteritis, bronchitis, conjunctivitis, and/or encephalitis in immunosuppressed and immunocompetent individuals. To better assess its genetic diversity, here we report near-chromosome-level draft genome assemblies for three geographically distinct isolates, quadrupling the number of genome assemblies available for the enigmatic fungal pathogen.

## ANNOUNCEMENT

The global prevalence of *Encephalitozoon intestinalis* infections is considered to be underestimated (1, 2), with recent studies suggesting 30% and 41% prevalence in individuals with Crohn’s disease and colorectal cancer (CRC), respectively (3, 4). To date, a singular *E. intestinalis* isolate has been sequenced, with the recent telomere-to-telomere assembly totaling 2.6 Mb across eleven complete chromosomes, resulting in minute, gene-dense linear molecules (5). Here, we used Illumina short-read sequencing to produce draft genome assemblies for three geographically distinct *E. intestinalis* isolates.

*E. intestinalis* isolates (ATCC 50507, ATCC 50603, and ATCC 50651; Table 1) were obtained from the American Type Culture Collection (ATCC) in early 2018. *E. intestinalis* spores were propagated in human foreskin fibroblasts in T150 flasks at 37°C, 5% CO_2_, with continuous culture in Dulbecco’s modified Eagle medium supplemented with 10% fetal bovine serum, 1% penicillin-streptomycin solution, and 2 mM l-glutamine (6). High molecular weight (HMW) genomic DNA (gDNA) was extracted from purified *E. intestinalis* spores (6) using the Lucigen MasterPure Complete DNA Purification Kit (LGC Biosearch Technologies, Teddington, Middlesex, UK) with an additional bead-beating step as described in reference 7. Library preparation was performed with the Nextera DNA Flex Kit (Illumina, San Diego, CA, USA), using 50 ng of HMW gDNA for each isolate. Sequencing was performed in-house on an Illumina MiniSeq instrument using a mid-throughput cartridge (151 bp paired-end reads).

**TABLE 1:** Sequencing and assembly statistics for three geographically distinct *Encephalitozoon intestinalis* isolates

	ATCC 50507	ATCC 50603	ATCC 50651
Metadata			
Location	New York	Georgia	California
Date	1994	1994	1993
Sequencing			
Total reads	8,558,306	6,968,272	7,652,156
Reads passing QC	6,539,490	5,369,796	6,042,318
Sequencing depth	361×	303×	346×
NCBI SRA	SRR24007516	SRR24007515	SRR24007514
Assembly			
# of contigs	28	32	28
%GC	41.34%	41.34%	41.34%
*N*_50_	180,619	136,524	146,790
Assembled bases	2,259,797	2,263,505	2,257,702
NCBI accessions	JBLUPJ000000000	JBLUPK000000000	JBLUPL000000000

Reads were quality-inspected with FASTQC v0.12.0 (https://www.bioinformatics.babraham.ac.uk/projects/fastqc/) and filtered with FASTP v0.23.2 (8) using a minimum *Q*-score of 30, a minimum read length of 125 bp, and “CTGCTCTTATACACATCT” as R1/R2 adapter sequences. Filtered reads were assembled with SPAdes v3.15.5 (9) using the “-k 33,55,77,99,111,121” extended k-mer suite and other parameters set to default, and contiguous sequences (contigs) smaller than 1 kb were discarded from the assemblies with process_fasta_sequences.py v0.2.2 (all custom scripts are available at https://github.com/PombertLab/Publication_scripts/tree/master/2025_E_intestinalis_isolates and archived at https://doi.org/10.5281/zenodo.17304204). Contaminants in the assemblies were identified and removed by performing BLAST homology searches (10) and by parsing the results with parseTaxonomizedBLAST.pl v0.2b using “microsporidians” as the organism of interest (-c sblastnames -n microsporidians). Filtered contigs were aligned and oriented to the *E. intestinalis* ATCC 50506 telomere-to-telomere genome (GCA_024399295.1) with orient_fastas_to_reference.py v0.3.5. Contigs that mapped to the same reference chromosome were checked for overlaps, and regions of overlap with 100% identity were merged with merge_contigs_by_overlap.py v0.2.0. Final assemblies were annotated as described in reference 6. Briefly, genes coding for proteins, ribosomal, and transfer RNAs were predicted using Prodigal v2.6.3 RNAmmer v1.2 (11) tRNAscan-SE v2.0.12 (12), for example, to fix missing start/stop codons, introns, or pseudogenes. Protein functions were transferred from previous *E. intestinalis* annotations (6) using annotate_by_bidirectional_blast.py v0.1.0.

## Data Availability

Annotated assemblies and raw sequencing data sets are deposited in the NCBI GenBank and Sequence Read Archive (SRA) databases, respectively (see Table 1).
